# Impact of sociodemographic and socioeconomic factors on functional and health-related quality of life outcomes 24 months after radical prostatectomy

**DOI:** 10.1007/s00432-025-06287-7

**Published:** 2025-09-09

**Authors:** M. Maas, K. Funk, V. Stühler, S. Walz, H. Bahlburg, J. Hennenlotter, J. Bedke, S. Aufderklamm, A. Stenzl, I. Tsaur, Steffen Rausch

**Affiliations:** 1 Department of Urology, University Hospital Tübingen, Eberhard Karls University, Hoppe-Seyler Str. 3, 72076 Tübingen, Germany; 2 Department of Pediatrics and Neonatology, University Hospital Tübingen, Eberhard Karls University, Tübingen, Germany; 3 Department of Urology, Marien Hospital Herne, Ruhr-University Bochum, Herne, Germany; 4 Department of Urology, Katharinenhospital Stuttgart, Stuttgart, Germany; 5 Department of Urology, General Hospital of Bregenz, Bregenz, Austria

**Keywords:** Radical prostatectomy, Patient-reported outcomes (PROs), Socioeconomic status (SES), Health-related quality of life (HRQoL), Psychosocial determinants

## Abstract

**Introduction and objectives:**

High socioeconomic status (SES) is associated with improved oncological outcomes across various cancer types, including prostate cancer. However, limited evidence exists regarding the impact of SES and lifestyle factors on patient-reported outcomes (PROs), including quality of life (QoL), health status (HS), and functional recovery following radical prostatectomy (RP).

**Materials and methods:**

We conducted a retrospective single-center analysis of 327 patients undergoing RP (177 open, 150 robotic-assisted) assessing pre- and postoperative functional outcomes (QoL, HS, erectile function, continence). PROs were evaluated 24 months postoperatively. Correlations with sociodemographic, socioeconomic (ISEI-based SES, marital status, occupational status, hometown size), and lifestyle factors (physical activity, BMI, mental stress) were analyzed.

**Results:**

Pathological features of locally advanced tumors correlated negatively with QoL and HS. Higher SES was significantly associated with improved continence, but not with QoL, HS, or erectile function. Pre-existing mental stress negatively affected both continence and HS. Regular physical activity correlated positively with QoL and HS. Multivariable regression confirmed these findings and identified mental stress, SES, partnership and physical activity as independent predictors of PROs.

**Conclusion:**

Beyond adverse tumor pathology, mental stress adversely impacts functional recovery and subjective health. In contrast, physical activity and a stable partnership correlate with better PROs. These findings may inform personalized patient counseling to increase postoperative satisfaction.

## Introduction

Prostate carcinoma is the most common malignancy in men worldwide, with the majority of cases diagnosed at a localized stage(Siegel et al. 2023). At this stage, standard treatment options include active surveillance, radical prostatectomy—either open (ORP) or robotic-assisted (RARP)—and radiotherapy (SEER 2023; Mottet 2023). Among these, surgery remains a commonly selected curative strategy, particularly in patients with intermediate risk disease.

While oncological outcomes after curative therapy are generally favorable in localized prostate cancer, the treatment imposes considerable physical and psychological burdens (Bourke et al. 2015). Despite being otherwise comparatively healthy, many patients experience a decline in quality of life (QoL), primarily due to erectile dysfunction (ED), urinary incontinence, fear of recurrence, and PSA-related anxiety (James et al. 2022; Carlsson et al. 2010). These issues have prompted calls for a more holistic approach to prostate cancer care—one that goes beyond an isolated focus on the organ itself and addresses both physical and psychosocial dimensions of patient well-being (Mottet 2023; Yiannopoulou et al. 2020).

Socioeconomic status (SES) is known to influence cancer incidence and mortality, with higher SES associated with better survival—largely due to greater health awareness, proactive screening behavior, improved access to health care providers and treatment adherence (Coughlin 2020; Cheng et al. 2009; Rapiti et al. 2009). Moreover, in various chronic conditions such as cardiovascular or renal disease, the combination of SES, sociodemographic characteristics, and lifestyle factors significantly impacts outcomes (Foster et al. 2021; Akyüz Özdemir et al. 2018; Zhang et al. 2020; Loef and Walach 2012). However, their role in influencing patient-reported outcomes (PROs) after prostatectomy remains underexplored.

This study aimed to assess the impact of sociodemographic, socioeconomic, and lifestyle factors on long-term postoperative PROs—specifically, QoL, perceived health status (HS), continence, and erectile function—24 months after radical prostatectomy.

We hypothesized that beyond established clinical predictors such as oncological and clinical features, factors such as SES, partnership status, and physical activity would show significant associations with subjective outcome measures. We expected higher SES to correlate with better PROs across all evaluated domains.

## Patients and methods

### Patient cohort

We retrospectively reviewed the institutional database in a comprehensive prostate cancer center for consecutive patients who underwent radical prostatectomy (RP)—either open (ORP) or robotic-assisted (RARP)—for localized prostate cancer between 2013 and 2016. All patients received a standardized, voluntary preoperative questionnaire assessing the outcome parameters evaluated in this study. Inclusion required complete questionnaire data at the time of surgery.

### Data sources

Sources of data included internal hospital records, rehabilitation reports, and patient-reported questionnaires administered preoperatively and 24 months postoperatively.

### Evaluated parameters

Parameters were grouped into three categories. Group 1: General data (age, surgical approach: ORP vs. RARP, oncological features (pT stage, pN status, Gleason score), and comorbidities, including perceived mental burden. Comorbidities were subclassified (e.g., cardiovascular, pulmonary, orthopedic, oncological, psychological), patients could appear in more than one category. Group 2**:** Sociodemographic and socioeconomic variables, including residence type, insurance type, marital status, number of children, living situation, familial problems, social participation, and employment status. Socioeconomic status was derived from occupation using ISCO-08 coding (International Standard Classification of Occupation 2008) and converted to the International Socio-Economic Index of Occupational Status (ISEI), ranging from 16 (low socioeconomic status) to 90 (high economic status). Analyses were performed with both dichotomized (> / ≤ median ISEI) and trichotomized SES groups (ISEI ≤ 30, 31–60, 61–90). Group 3: Lifestyle variables, including alcohol and nicotine use, physical activity, dietary habits, and body mass index (BMI).

### Outcome parameters

Subjective quality of life (QoL), perceived health status (HS), continence, and erectile function were assessed preoperatively and at 24 months postoperatively using validated questionnaires. QoL and HS were measured by single-item questions from the EORTC QLQ-C30 on a 0–7 Likert scale (0 = very poor, 7 = excellent). Continence was assessed using the ICIQ, consisting of three Likert-scaled items (total score 0–21); incontinence was categorized as mild (1–5) or severe (≥ 11). Erectile function was measured with the IIEF-5 (score range 0–25), where higher scores indicate better function.

### Statistical analysis

Nominal variables were analyzed using univariate tests (Wilcoxon, Mann–Whitney U, Kruskal–Wallis). Bivariate adjustment was applied for continuous variables; correlation coefficients and linear regression (R-values) were used to examine associations. Ordinal variables were analyzed via contingency tables with likelihood ratio testing. Variables with significant univariate correlations were further analyzes using multivariable linear regression, adjusting for age, pT stage, pN status, Gleason score, and surgical technique. Individual models were calculated for each patient-reported outcome. A *p*-value < 0.05 was considered statistically significant.

### Ethics and data privacy

The study was approved by the Ethics Committee of the University of Tübingen (711/2021BO2). Data were pseudonymized and stored under unique patient identifiers.

## Results

A total of 327 patients were eligible for the study, of whom 177 (54.1%) underwent open and 150 (45.9%) robotic-assisted radical prostatectomy. The median age at surgery was 65.1 years (range 45.5–83.5, 95% CI 64.1–66.5). The most common tumor stage was pT2 (73.1%, 236 patients), and 89.4% had pN0 status (287 patients). Gleason Score 7a was the most frequent score (47.1%, 153 patients). Comorbidities were reported in 64.8% (212 patients), with hypertension being most prevalent (47.7%). Clinical characteristics are detailed in Table 1.

**Table 1:** Clinical and pathological features of the cohort

Patient collective		
	n	
Patients	327	100%
*Surgical method*		
Open radical prostatectomy	177	54.1%
DaVinci-assisted radical prostatectomy	150	45.9%
*Age*	*327/327 available*	
Median	65.11 years	95% CI: 64.09 – 66.53 years
Range	54.51 – 83.47 years	
*pT-stage*	*323/327 available*	
pT1c	2	0.61%
pT2a	40	12.38%
pT2b	7	2.17%
pT2c	189	58.51%
pT3a	45	13.93%
pT3b	40	12.38%
*pN-status*	*321/327 available*	
pN0	287	89.41%
pN1	34	10.59%
*Gleason score*	*325/327 available*	
Gleason score 6	35	10.77%
Gleason score 7a	153	47.08%
Gleason score 7b	81	24.92%
Gleason score 8	23	7.08%
Gleason score 9	33	10.15%
*Pre-existing conditions*	*327/327 available*	
No pre-existing conditions	115	35.17%
Pre-existing conditions	212	64.83%
Arterial hypertension	156	47.71%
Cardiac pre-existing conditions	33	10.09%
Orthopedic pre-existing conditions	31	9.48%
Oncological pre-existing conditions	28	8.56%
D. mellitus type II	23	7.03%
Pulmonary pre-existing conditions	17	5.20%
Mental health pre-existing conditions	8	2.45%
Other pre-existing conditions	63	19.27%
*Perceived mental burdenD*	*187/325 available*	
Pre-existing	23	12.30%
PCa-related	27	14.44%

Most patients lived in urban areas with > 10,000 inhabitants (65.4%), and 70.7% had public health insurance (222 patients). Marital status was predominantly married/widowed (85.6%), 14.4% were single or divorced. 6.9% (18 patients) lived alone. A total of 88.3% (233 patients) had children (mean 1.85 children). Family issues were reported in 5.2% (9 patients), and social participation was impaired in 3.4% (6 patients).

At the time of surgery, 42.3% (112 patients) were employed (75% salaried, 25% self-employed), while 57.7% (153 patients) were retired or in partial retirement. The mean ISEI score was 55.4. Based on ISEI, 9.4% were assigned to low, 47.7% to intermediate, and 42.9% to high socioeconomic status (see Table 2 for detailed overview of sociodemographic and socioeconomic characteristics).

**Table 2:** Sociodemographic and socioeconomic features of the cohort

Sociodemographic characteristics		
*Place of residence*	*327/327 available*	
Rural community (< 5000 inhabitants)	42	12.84%
Small town (5000–19,999 inhabitants)	71	21.71%
Medium-sized city (20,000–99,999 inhabitants)	168	51.38%
Large city (> 100,000 inhabitants)	46	14.07%
*Insurance status*	*314/327 available*	
Public health insurance	222	70.70%
Private Insurance	92	29.30%
*Marital status*	*271/327 available*	
Single	17	6.27%
Married	228	84.13%
Divorced	22	8.12%
Widowed	4	1.48%
Children	*264/327 available*	
0	31	11.74%
1	55	20.83%
2	119	45.08%
3	42	15.91%
4	15	5.68%
5	2	0.76%
Average number of children	1.85	
*Family issues*	*172/327 available*	
Existent	9	5.23%
Not existent	163	94.77%
*Social participation*	*175/327 available*	
Not impaired	169	96.57%
Impaired	6	3.43%
*Employment situation*	*265/327 available*	
Partial retirement or retired	153	57.74%
Employed	112	42.26%
Salary-employed	84	75.00% (of employed patients)
Self-employed	28	25.00% (of employed patients)
*International socio-economic index of occupational status (ISEI)*	*266/327 available*	
Median ISEI	55.39	
Low ISEI (16–30)	25	9.40%
Intermediate ISEI (31–60)	127	47.74%
High ISEI (61–90)	114	42.86%

Regarding lifestyle, 50.2% reported occasional alcohol use, 88.5% were non-smokers (278 patients), and 58.7% (108 patients) engaged in regular physical activity. Only 1.7% (5 patients) followed a strictly vegetarian diet. According to WHO BMI categories, 78.3% were normal weight or overweight, with overweight being most frequent (48.4%) (Table 3 summarizes reported lifestyle factors).

**Table 3:** Lifestyle factors of the cohort

Lifestyle factors		
*Body-index*	*315/327 available*	
*Mean BMI*	26.97 kg/m^2^	
Underweight (BMI < 18.5 kg/m^2^)	2	0.63%
Normal weight (18.5–24.9 kg/m^2^)	94	29.84%
Overweight (25–29.9 kg/m^2^)	152	48.25%
Obesity class I (30–34.9 kg/m^2^)	61	19.37%
Obesity class II (35–39.9 kg/m^2^)	5	1.59%
Obesity class III (> 40 kg/m^2^)	1	0.32%
*Diet*	*298/327 available*	
Vegetarian	5	1.68%
No restrictions	293	98.32%
*Physical activity*	*184/327 available*	
None	23	12.50%
Light	53	28.80%
Regular	108	58.70%
*Alcohol consumption*	*305/327 available*	
Never—rarely	93	30.49%
Occasionally	153	50.16%
Moderate—regular	59	19.34%
*Nicotine consumption*	*314/327 available*	
Consumption	36	11.46%
No consumption	278	88.54%

**Fig. 1:**
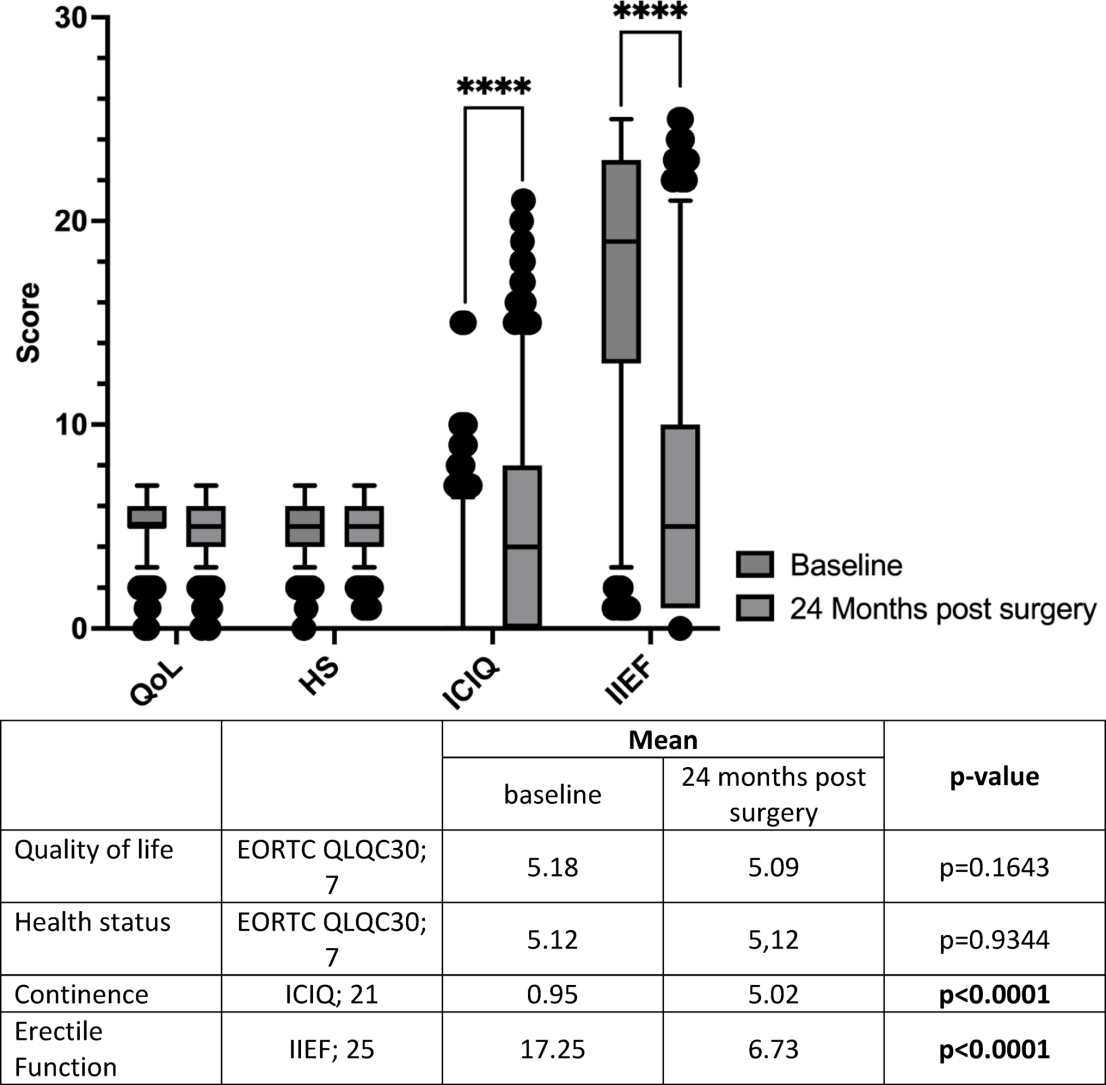
Comparison of patient-reported outcomes from baseline (prior to surgery) and 24 months after radical prostatectomy. No significant differences were observed in QoL or health status between baseline and 24 months postoperatively, while continence and erectile function declined significantly

No significant differences were observed in QoL or health status between baseline and 24 months postoperatively (QoL: 5.18 vs. 5.09, *p* = 0.1643; HS: 5.12 vs. 5.12, *p* = 0.9344). In contrast, continence and erectile function declined significantly (ICIQ: 0.95 vs. 5.02, *p* < 0.0001; IIEF: 17.25 vs. 6.73, *p* < 0.0001) (Fig. 1).

### Quality of life

**Fig. 2:**
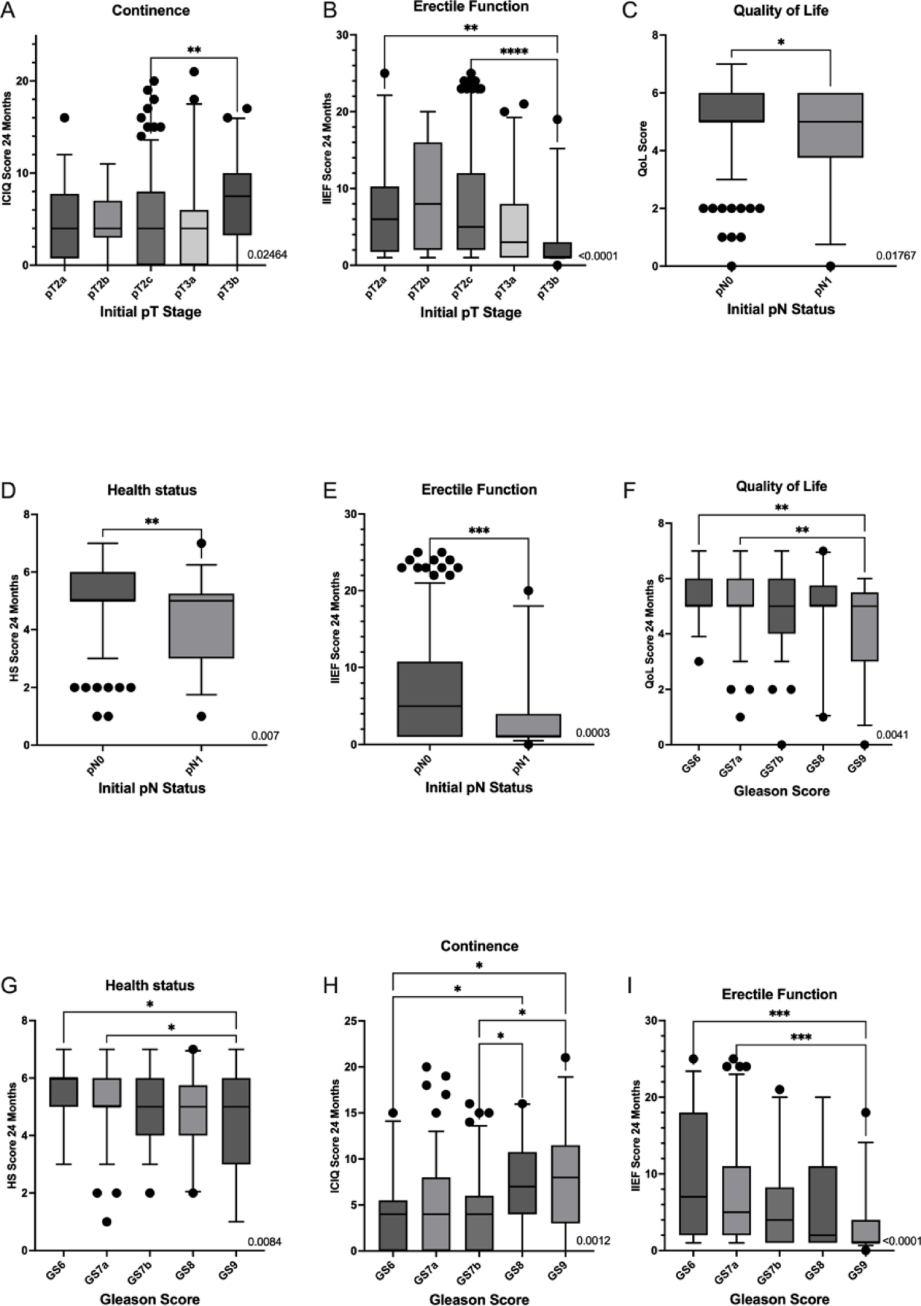
Association of tumor-related pathological parameters with patient-reported outcomes 24 months post surgery (only significant results are shown). **A** Association between pT stage and continence after 24 months illustrating worse outcome in patients with pT3b tumors; **B** Association of pT stage and erectile function after 24 months illustrating worse outcome in patients with pT3b tumors; **C** Association between nodal status and quality of life at 24 months, indicating lower QoL in patients with pN1 disease; **D** Association between pN status and quality of life at 24 months, indicating impaired perceived health in patients with pN1 disease; **E** Association between pN status and erectile function after 24 months indicating worse erectile function in patients with pN1 disease; **F** Association of Gleason score and QoL after 24 months indicating worse outcome in patients with higher Gleason scores; **G** Association of Gleason score and HS after 24 months indicating worse outcome in patients with higher Gleason scores; **H** Association of Gleason score and continence after 24 months indicating worse outcome in patients with higher Gleason scores; **I** association of Gleason score and erectile function after 24 months indicating worse outcome in patients with higher Gleason scores

**Fig. 3:**
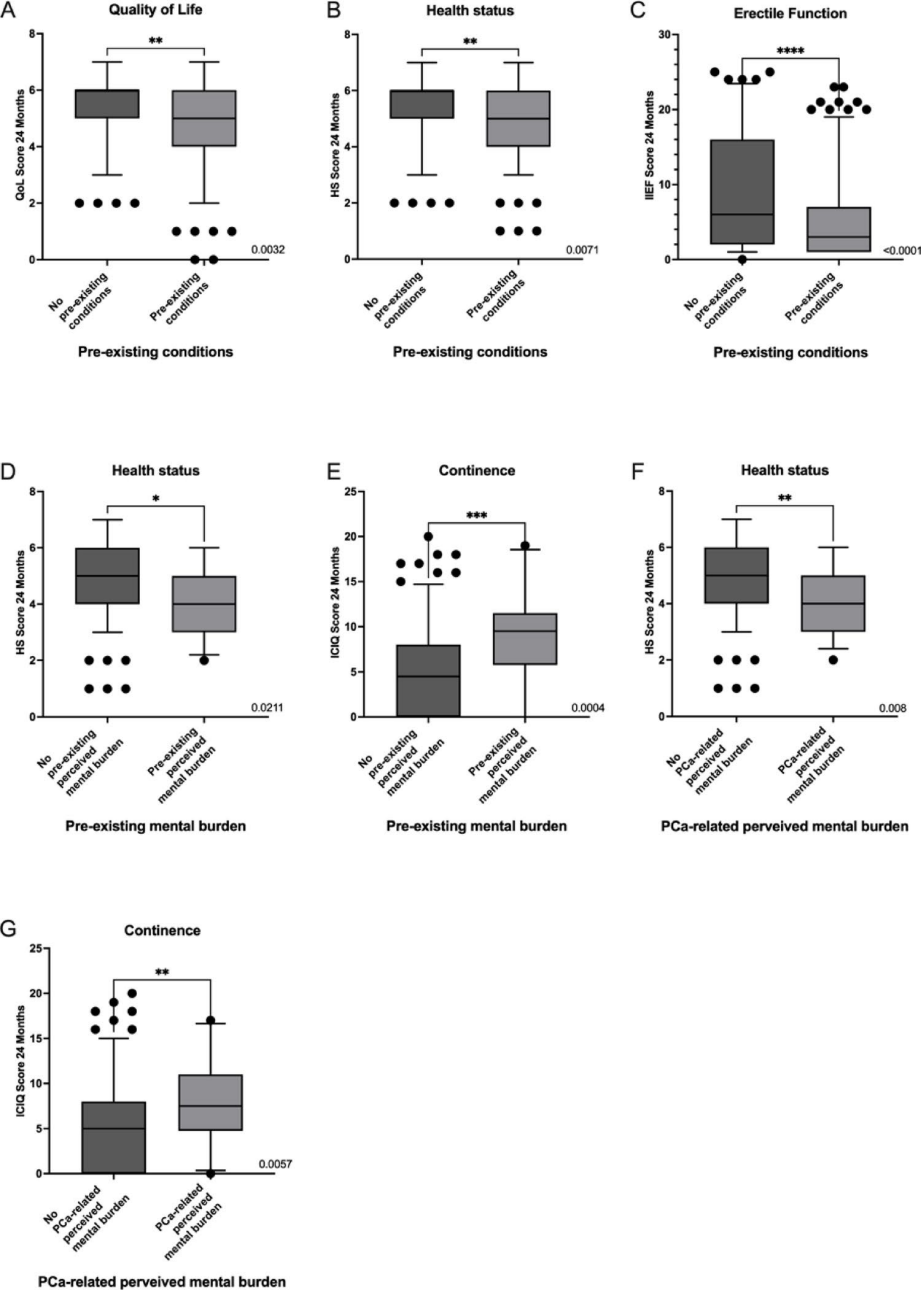
Association of pre-existing conditions and perceived mental burden with patient-reported outcomes 24 months post surgery (only significant results are shown). **A** Association of pre-existing conditions and QoL after 24 months indicating impaired QoL in patients with pre-existing conditions; **B** Association of pre-existing conditions and HS after 24 months indicating impaired perceived HS in patients with pre-existing conditions; **C** Association of pre-existing conditions and erectile function after 24 months indicating worse erectile function in patients with pre-existing conditions; **D** Association of pre-existing mental burden and HS after 24 months indicating impaired perceived HS in patients with pre-existing mental burden; **E** Association of pre-existing mental burden and continence after 24 months indicating impaired continence in patients with pre-existing mental burden; **F** Association of PCa- related perceived mental burden and HS after 24 months indicating impaired perceived HS in patients with perceived PCa- related mental burden; **G** PCa- related perceived mental burden and continence after 24 months indicating impaired continence in patients with perceived PCa- related mental burden

Correlation analysis of influencing factors on QoL after 24 months revealed a significant lower QoL at 24 months in patients with lymph node metastases (4.471 vs. 5.129, *p* = 0.0177), higher Gleason scores (overall *p* = 0.0041; Gleason 6 vs. 9: *p* = 0.0096; 7a vs. 9: *p* = 0.0066) (see Fig. 2), and comorbidities (4.910 vs. 5.325, *p* = 0.0032) (see Fig. 3 and Table 4). Furthermore, unpartnered individuals reported worse QoL (4.774 vs. 5.162, *p* = 0.0474). Regular physical activity was associated with higher QoL (5.106 vs. 4.348, *p* = 0.0069) (Table 5). In multivariable linear regression, none of the influencing factors were independently associated with QoL after 24 months. Partnership and physical activity showed positive trends but did not reach statistical significance (*p* = *0.29 and p* = *0.15*). Multivariable regression results are summarized in Table 6.

**Table 4:** Correlation analysis

	Quality of Life		Health status		Continence (ICIQ)		Erectile function (IIEF)	
	Spearman’s r	*p-*value	Spearman’s r	*p-*value	Spearman’s r	*p-*value	Spearman’s r	*p-*value
*Group 1: Clinical and oncological parameters*								
Age	− 0.151	0.0542	**− 0.1499**	0.0998	0.0078	0.3523	** − 0.3305**	** < 0.0001**
pT-Stage	− 0.05013	0.3911	− 0.0814	0.161	**0.1186**	**0.0434**	** − 0.2584**	** < 0.0001**
pN-Status	** − 0.1324**	**0.0173**	** − 0.1496**	**0.0075**	0.1028	0.0672	** − 0.2013**	**0.0004**
Gleason Score	** − 0.1742**	**0.0034**	** − 0.1519**	**0.012**	0.0571	**0.0138**	** − 0.2284**	** < 0.0001**
Pre-existing conditions	** − 0.1635**	**0.0026**	** − 0.1491**	**0.0071**	0.0739	0.1841	** − 0.2644**	** < 0.0001**
Arterial Hypertension	** − 0.1201**	**0.0272**	− 0.0808	0.1448	0.0792	0.1545	** − 0.1973**	**0.0005**
Cardiac pre-existing conditions	** − 0.1113**	**0.043**	** − 0.1149**	**0.038**	− 0.0001	0.9881	** − 0.1593**	**0.0048**
Orthopedic pre-existing conditions	− 0.0704	0.1995	− 0.0554	0.3168	0.0711	0.2016	** − 0.1391**	**0.0137**
Oncological pre-existing conditions	− 0.0622	0.2556	− 0.0791	0.1532	0.0518	0.3519	** − 0.1652**	**0.0034**
D. mellitus Type II	− 0.0705	0.212	− 0.0529	0.3525	0.0449	0.4323	− 0.1123	0.053
Pulmonary pre-existing conditions	− 0.07024	0.2009	− 0.0738	0.1829	0.0467	0.4011	− 0.0110	0.8461
Mental Health pre-existing conditions	− 0.06937	0.2081	− 0.0697	0.2083	0.0433	0.4367	− 0.0207	0.7139
Other pre-existing conditions	** − 0.1322**	**0.0161**	− 0.1043	0.0597	0.0874	0.1161	** − 0.1309**	**0.0204**
Pre-existing perceived mental burden	− 0.1266	0.0753	** − 0.1687**	**0.0084**	**0.2550**	**0.0006**	− 0.0253	0.7362
PCa-related perceived mental burden	− 0.1283	0.0809	** − 0.1931**	**0.0217**	**0.2018**	**0.0062**	− 0.0552	0.4605
*Group 2: Sociodemographic and socioeconomic parameters*								
Place of residence	− 0.4592	0.8277	− 0.0513	0.9081	0.0224	0.5543	− 0.0719	0.4241
Insurance status	0.0749	0.1629	0.1087	0.0545	− 0.0692	0.2228	− 0.0385	0.5054
Marital status	** − 0.1212**	**0.0321**	** − 0.1201**	**0.0287**	0.0607	0.2502	− 0.0114	0.9316
Family issues	− 0.1209	0.1106	− 0.0626	0.4127	0.1091	0.1563	**0.1550**	**0.0465**
Social participation	− 0.0470	0.5272	− 0.0440	0.5643	0.1262	0.0968	− 0.1915	0.8034
Employment situation	0.1015	0.0779	− 0.0827	0.1647	− 0.0832	0.1747	**0.3427**	** < 0.0001**
International socio-economic index of occupational status (ISEI)	0.0527	0.1474	0.0507	0.1707	** − 0.1451**	**0.0003**	− 0.0578	0.3874
*Group 3: Lifestyle parameters*								
BMI	− 0.1025	0.2621	− 0.1033	0.2875	0.1186	0.0908	** − 0.1427**	**0.0020**
Diet	− 0.0078	0.8937	− 0.0489	0.3986	− 0.1044	0.8254	− 0.0655	0.2670
Physical activity	**0.1724**	**0.0198**	**0.1542**	**0.0174**	0.0162	0.2806	0.0030	0.7021
Alcohol consumption	− 0.0135	0.7248	0.0112	0.4638	− 0.0038	0.4638	0.0094	0.9436
Nicotine consumption	− 0.0301	0.7127	− 0.0643	0.7089	− 0.0783	0.7089	**0.1527**	**0.0080**

Statistically significant correlations are marked in bold

**Table 5:** Statistically significant differences in the evaluated QoL Score, HS score, ICIQ and IIEF

Parameter	Mean	*p*-value
*Quality of Life 24 months*		
pN0 vs. pN +	5.129 vs. 4.471	*p* = 0.0177
Gleason score	Gleason score 6: 5.459 Gleason score 7a: 5.237 Gleason score 7b: 4.952 Gleason score 8: 4.850 Gleason score 9: 4.152	*p* = 0.0041 Multiple comparisons: GS 6 vs. GS 9, *p* = 0.0096 GS 7a vs. GS 9, *p* = 0.0066
No pre-existing conditions vs. pre-existing conditions	5.325 vs. 4.910	*p* = 0.0032
Unpartnered vs. partnered	4.674 vs. 5.149	*p* = 0.0386
No physical activity vs. physical activity	4.348 vs. 5.106	*p* = 0.0069
*Health status 24 months*		
pN0 vs. pN +	5.188 vs. 4.559	*p* = 0.007
Gleason score	Gleason score 6: 5.405 Gleason score 7a: 5.275 Gleason score 7b: 5.107 Gleason score 8: 4.240 Gleason score 9: 3.699	*p* = 0.0084 Multiple comparisons: GS 6 vs. GS 9, p = 0.0352 GS 7a vs. GS 9, p = 0.0132
No pre-existing conditions vs. pre-existing conditions	5.348 vs. 4.995	*p* = 0.0071
Unpartnered vs. partnered	4.744 vs. 5.162	*p* = 0.0474
No physical activity vs. physical activity	4.261 vs. 5.112	*p* = 0.0042
No pre-existing perceived mental burden vs. pre-existing perceived mental burden	4.975 vs. 4.348	*p* = 0.0211
No PCa-related perceived mental burden vs. PCa-related perceived mental burden	4.994 vs. 4.333	*p* = 0.0080
*Continence 24 months*		
pT-stage	pT2a: 4.950 pT2b: 4.857 pT2c: 4.674 pT3a: 4.818 pT3b: 7.200	*p* = 0.0246 Multiple comparisons: pT2c vs. pT3b, *p* = 0.0099
Gleason score	Gleason score 6: 4.000 Gleason score 7a: 4.921 Gleason score 7b: 4.060 Gleason score 8: 7.350 Gleason score 9: 7.636	*p* = 0.0012 Multiple comparisons: GS 6 vs. GS 8, *p* = 0.0488 GS 6 vs. GS 9, *p* = 0.0441 GS 7b vs. GS 8, *p* = 0.02588 GS 7b vs. GS 9, *p* = 0.0155
No pre-existing perceived mental burden vs. pre-existing perceived mental burden	5.210 vs. 8.955	*p* = 0.0004
No PCa-related perceived mental burden vs. PCa-related perceived mental burden	5.333 vs. 8.000	*p* = 0.0057
ISEI low vs. ISEI high	5.748 vs. 4.264	*p* = 0.0189
*Erectile function 24 months*		
Age (> median vs. ≤ median)	8.439 vs. 4.747	*p* < 0.0001
pT-stage	pT2a: 7.632 pT2b: 9.571 pT2c: 7.527 pT3a: 5.119 pT3b: 3.184	*p* < 0.0001 Multiple comparisons: pT2c vs. pT3b, *p* < 0.0001 pT2a vs. pT3b, *p* = 0.0020
pN0 vs. pN +	7.043 vs. 3.379	*p* = 0.0003
Gleason score	Gleason score 6: 9.771 Gleason score 7a: 7.411 Gleason score 7b: 5.833 Gleason score 8: 5.737 Gleason score 9: 2.969	*p* < 0.0001 Multiple comparisons: GS 6 vs. GS 9, *p* = 0.0001 GS 7a vs. GS 9, *p* = 0.0002
No pre-existing conditions vs. pre-existing conditions	9.264 vs. 5.371	*p* < 0.0001
No existing family issues vs. existing family issues	5.930 vs. 10.63	*p* = 0.0450
Employed vs. retired/ partial employed	8.890 vs. 4.696	*p* < 0.0001
Non-obese vs. obese	7.201 vs. 5.138	*p* = 0.0285
No nicotine consumption vs. nicotine consumption	6.502 vs. 8.794	*p* = 0.0076

**Table 6:** Summary of multivariable regression models for all patient-related outcomes 24 months after radical prostatectomy

Predictor variable	QoL (p)	Health status (p)	Continence (p)	Erectile function (*p*)
Age (years)	0.0892*	0.7152	0.377	< 0.0001*
*pT stage*				
pT2a	0.9161	0.3423	0.0890	0.5984
pT2b	0.1677	0.1289	0.5051	0.2613
pT2c	0.9109	0.8030	0.9270	0.2085
pT3a	0.4107	0.9345	0.7532	0.1644
pT3b	0.6323	0.2954	0.3782	0.0011*
pN status	0.963	0.9086	0.5545	0.8802
*Gleason score*				
GS6	0.4578	0.5223	0.6127	0.6752
GS7a	0.4725	0.1585	0.2223	0.0474*
GS7b	0.3216	0.2933	0.6701	0.0890
GS8	0.1015	0.0507	0.5766	0.7776
GS9	0.0055*	0.0048*	0.1129	0.0417
Surgical technique	0.1724	0.7965	0.0432*	0.8431
Mental stress	–	0.2261	0.0298*	–
Tumor-associated stress	–	0.0215*	0.1193	–
Physical activity	0.1461	0.0257*	–	–
Partnership	0.2942	0.4359	–	–
Comorbidities	0.4736	0.4282	0.2342	0.0083*
SES	–	–	0.0125*	–
BMI	–	–	–	0.0886
Nicotine consumption	–	–	–	0.2436
Model R2	0.182	0.2237	0.209	0.292

Quality of life (QoL), general health status, urinary continence (ICIQ), and erectile function (IIEF-5). Significant *p*-values (*p* < 0.05) are marked in bold and with an asterisk (*). ‘ns’ indicates no statistically significant association; ‘–’ indicates that the variable was not included in the model

### Health status

**Fig. 4:**
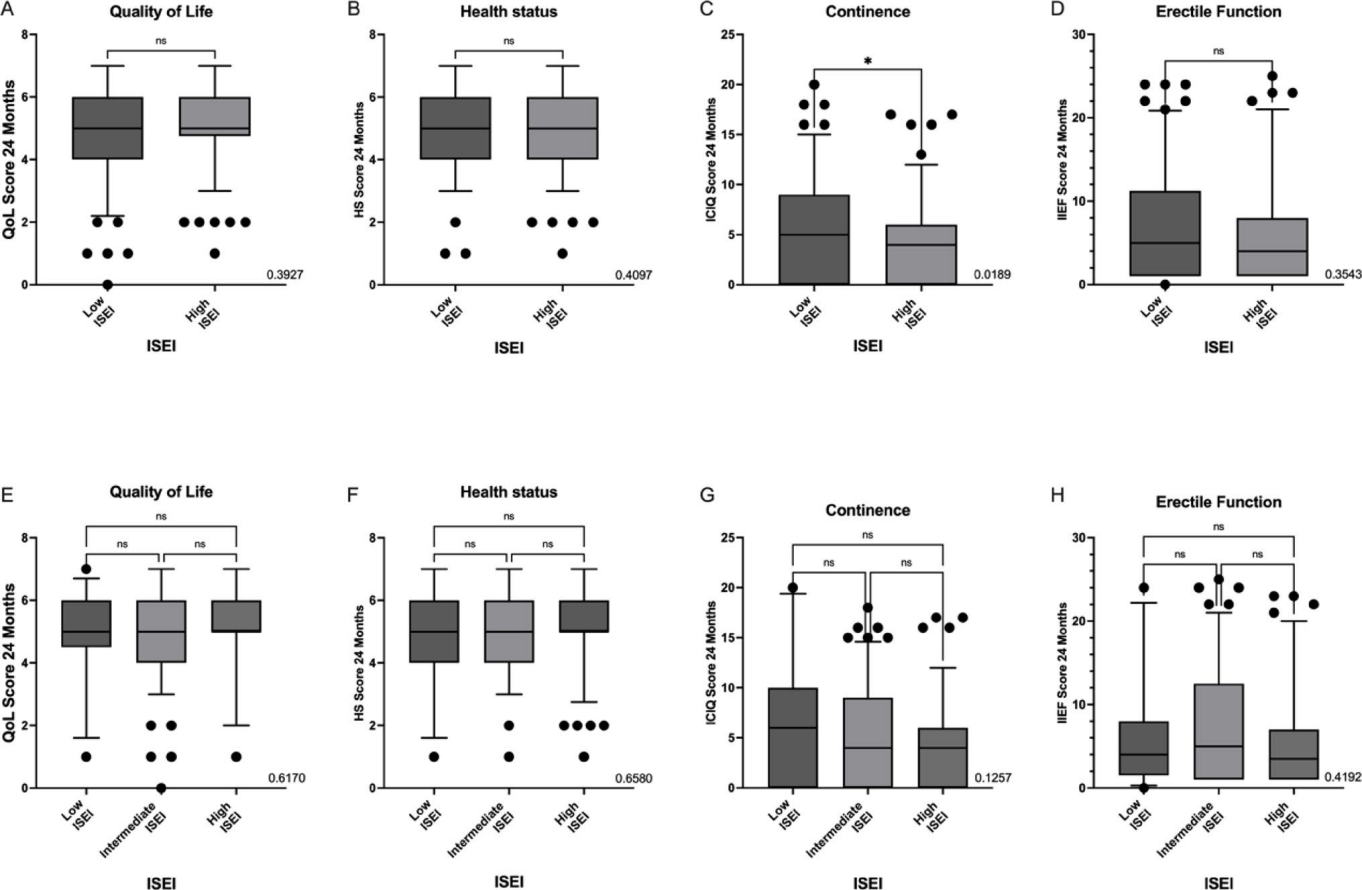
Association of SES and patient-reported outcomes 24 months post surgery. **A**–**D** dichotomized analyses. **A** Comparison of low vs. high SES showing no significant differences in perceived QoL after 24 months; **B** Comparison of low vs. high SES showing no significant differences in perceived HS after 24 months; **C** Comparison of low vs. high SES indicating a better continence in patients with high SES after 24 months; **D** Comparison of low vs. high SES showing no significant differences in erectile function after 24 months. **E**–**H** trichotomized analyses. **E** Comparison of low vs. intermediate vs. high SES showing no significant differences in perceived QoL after 24 months; **F** Comparison of low vs. intermediate vs. high SES showing no significant differences in perceived HS after 24 months; **G** Comparison of low vs. intermediate vs. high SES showing no significant differences in continence after 24 months; **H** Comparison of low vs. intermediate vs. high SES showing no significant differences in erectile function after 24 months

Analyses of perceived HS showed similar correlations. Lower scores were observed in patients with nodal metastases, higher Gleason scores (overall *p* = 0.0084), and comorbidities (see Figs. 3 and 4). Significant differences were found between Gleason 6 and 9 (5.405 vs. 3.699, *p* = 0.0352) and between 7a and 9 (5.275 vs. 3.699, *p* = 0.0132) (see Fig. 2). Patients with perceived mental stress—pre-existing or tumor-associated—reported significantly lower HS (4.348 and 4.333 vs. 4.975 and 4.994; *p* = 0.0211 and *p* = 0.0080) (see Fig. 3). Positive correlations were again noted for partnership and physical activity. Multivariable linear regression confirmed physical activity as an independent positive predictor (*p* = 0.0257), while perceived tumor-related stress emerged as an independent negative factor (*p* = 0.0215; see Table 6).

### Continence

Higher ICIQ scores (reporting worse continence) were associated with advanced T-stage (overall *p* = 0.0246; in multiple comparisons only significant in pT2c vs. pT3b, *p* = 0.0099) and higher Gleason scores (overall *p* = 0.0012), particularly between Gleason 6 and 8 or 9 (Mean: 4.000 vs. 7.350, *p* = 0.0488 and Mean: 4.000 vs. 7.636, *p* = 0.0441) and Gleason 7 b and 8 or 9 (Mean: 4.060 vs. 7.350, *p* = 0.02588 and Mean: 4.060 vs. 7.636, *p* = 0.0155). Mental stress (both pre-existing and tumor-related) was strongly linked to poorer continence (8.955 vs. 5.210, *p* = 0.0004; 8.000 vs. 5.333, *p* = 0.0057) (see Fig. 3). Patients with higher SES had better continence outcomes (ISEI high vs. low: 4.264 vs. 5.748, *p* = 0.0189) (see Fig. 4). Lifestyle factors showed no significant correlation with continence. Multivariable analysis confirmed mental stress and socioeconomic status as independent predictors (*p* = 0.0298 and 0.0125; see Table 6).

### Erectile function

Lower IIEF scores (indicating worse erectile function) were associated with higher age (Mean IIEF: 4.747 vs. 8.439, *p* < 0.0001), higher tumor stage, nodal metastases, and higher Gleason scores (all *p* < 0.0001) (see Fig. 2). Comorbidities (Mean IIEF: 5.371 vs. 9.264, *p* < 0.0001), retirement status (Mean IIEF: 4.696 vs. 8.890, *p* < 0.0001), and obesity (Mean IIEF: 5.138 vs. 7.201, *p* = 0.0285) also correlated negatively (see Fig. 2). Paradoxically, smokers and patients reporting family issues had higher IIEF scores than their counterparts (nicotine: 8.794 vs. 6.502, *p* = 0.0076; family issues: 10.63 vs. 5.930, *p* = 0.0450). In multivariable analysis, BMI remained just above the threshold of statistical significance, while nicotine consumption was no longer significant; see Table 6).

### Detailed analysis of socioeconomic status

Given the initial hypothesis, we examined the impact of socio-economic status, as determined by the ISEI, on the evaluated parameters in more detail. In the dichotomized analysis, no significant differences between patients with high socio-economic status and those with low socio-economic status regarding of QoL (Mean 5.092 vs. 4.943, *p* = 0.3927), HS (Mean 5.154 vs. 5.033, *p* = 0.4097), and erectile function (Mean 6.294 vs. 7.033, *p* = 0.3543) were observed. Higher SES was however significantly associated with better continence (ICIQ: 4.264 vs. 5.748, *p* = 0.0189). In trichotomized analysis, no significant differences were observed in any parameter (QoL: 5.096 vs. 4.944 vs. 5.080, *p* = 0.7423; HS: 5.175 vs. 5.055 vs. 4.960, *p* = 0.6170; IIEF: 6.019 vs. 7.328 vs. 5.920, *p* = 0.4192; ICIQ: 4.226 vs. 4.724 vs. 5.817 *p* = 0.1257). Results are graphically presented in Fig. 4. In the multivariable analysis treating SES as a continuous variable, higher SES was confirmed as an independent predictor of better continence 24 months after surgery (see Table 6).

## Discussion

In the present study, we evaluated the influence of sociodemographic, socioeconomic, and lifestyle factors on patient-reported outcomes (PROs) 24 months after radical prostatectomy. Our findings confirm that classical pathological risk factors—namely lymph node metastases and high Gleason scores—are significantly associated with impaired subjective (quality of life, QoL; health status, HS) and functional (continence, erectile function) outcomes. These results align with prior studies demonstrating a negative impact of higher tumor burden — although assessed using different parameters than in our study — on functional recovery and health-related quality of life (Sanda et al. 2008; Punnen et al. 2015).

Beyond these oncological variables, perceived mental stress—both pre-existing and tumor-associated—emerged as a relevant determinant not only of perceived health status but, notably, also of continence. While mental health is increasingly recognized as a relevant factor in cancer survivorship, few studies have directly linked pre-existing psychological distress to objective functional outcomes such as continence (Onitilo et al. 2006; Ayres et al. 1994; Emanu et al. 2016). Our findings thus suggest that integrating psychosocial screening into perioperative care may be beneficial for improving patient-centered outcomes.

Additionally, regular physical activity and the presence of a stable partnership were positively associated with QoL and HS, supporting the relevance of psychosocial and behavioral support in survivorship care. These observations align with previous research showing that physical activity promotes recovery and enhances perceived well-being following prostate cancer treatment (Bourke et al. 2014). Similarly, the protective effect of social support—particularly through stable partnerships or close relationships—has been identified as a crucial determinant of patient satisfaction and psychological resilience in the context of cancer survivorship (Hagedoorn et al. 2008).

Traditional lifestyle variables such as smoking, alcohol use, and BMI showed only limited associations with postoperative outcomes in our analysis. Notably, a paradoxical positive correlation between nicotine use or reported family problems and erectile function was observed. These findings warrant cautious interpretation. While metabolic health—partly reflected by BMI—has been previously linked to erectile dysfunction in various populations, a positive association between nicotine use and erectile function appears questionable, as it contrasts with established evidence. Smoking has consistently been associated with impaired erectile function via vascular and endothelial mechanisms, and increased BMI has been linked to a higher prevalence of erectile dysfunction (He et al. 2007; Bacon et al. 2006; Liu et al. 2023; Pizzol et al. 2020). These unexpected associations may be attributable to residual confounding or reporting bias, which is further supported by their loss of significance in the multivariable model.

Our central hypothesis was that – in addition to well-established clinical and pathological predictors—sociodemographic, socioeconomic, and lifestyle factors would independently influence long-term functional and health-related quality-of-life outcomes following radical prostatectomy. This assumption was based on evidence from chronic disease models and oncological research, where factors such as social support, health awareness, and individual behavioral patterns have been shown to modulate both objective outcomes and patient satisfaction (Stringhini et al. 2017; Mackenbach et al. 2008). Given the high survival rates in localized prostate cancer and the shift toward survivorship care, identifying modifiable predictors of patient-perceived outcome dimensions is of particular clinical importance (Crawford-Williams et al. 2018). Our study aimed to contribute to this understanding by exploring multidimensional influences beyond the disease itself.

A distinctive strength of our study lies in the comprehensive evaluation of determinants beyond clinical-pathological variables. Specifically, we assigned an individual socioeconomic status (SES) to each patient based on occupational classification using ISCO-08 codes, converted into the International Socio-Economic Index of Occupational Status (ISEI). This enabled a differentiated, quantitative analysis of socioeconomic influence at the individual level in a real-world cohort—an approach that, to our knowledge, has rarely been implemented with this level of detail in prostate cancer outcome research.

Despite this detailed stratification, we observed only a limited impact of SES on patient-reported outcomes: While higher SES were significantly associated with better continence in the binary stratification model, no significant association was found for QoL, HS, or erectile function. Moreover, significance for continence disappeared in the categorized analysis, suggesting a less consistent influence of SES on broader subjective outcome measures post-prostatectomy. However, when modeled as a continuous variable in the multivariable analysis, SES strengthened its role as an independent predictor of better postoperative continence, possibly reflecting greater adherence to postoperative physiotherapy in patients with higher SES.

Our results are partly inconsistent with existing evidence. For instance, Karakiewicz et al. reported significantly worse urinary and sexual function in low-SES patients after prostatectomy in a U.S. cohort (Karakiewicz et al. 2008). Similarly, Mahal et al. demonstrated that socioeconomic disadvantage was associated with poorer health-related quality of life across multiple domains in patients with localized prostate cancer (Mahal et al. 2014). Both studies reflect healthcare environments characterized by variable access to rehabilitation and follow-up services.

In contrast, the more attenuated and domain-specific associations observed in our cohort may be explained by structural features of the German healthcare system. Universal health coverage, standardized follow-up protocols, and broad access to inpatient rehabilitation likely reduce SES-related disparities, particularly with respect to global outcome measures such as QoL and perceived health status. The observed association between higher SES and improved continence may nonetheless reflect behavioral or structural mediators, including greater utilization of physiotherapy, higher health literacy, or stronger engagement with supportive care among patients with higher SES. Supporting this, a Swedish registry study showed that men with lower educational and income levels experienced significantly worse functional outcomes and quality of life after radical prostatectomy, despite a universal healthcare setting (Carlsson et al. 2016). Together, these findings highlight that even in egalitarian systems, SES may influence recovery trajectories through indirect pathways.

Our study includes several limitations. It is single-center and conducted in Germany, limiting generalizability of the findings to other populations or healthcare systems. The retrospective design carries an inherent risk of unmeasured confounding. Changes in SES or lifestyle post-surgery—both of which may influence long-term outcomes—could not be assessed. Although the ISEI is a validated tool for classifying occupational status, it may not fully reflect other relevant social dimensions such as education, income, or access to informal support networks. Additionally, participation in the follow-up survey was voluntary, introducing a possible selection bias. Patients who were more health-conscious or satisfied with their outcomes may have been more likely to respond, which could skew the observed associations.

Another limitation concerns the sample size of specific subgroups. Particularly within SES strata, limited numbers may have reduced statistical power and obscured potentially meaningful differences. The use of self-reported instruments introduces subjectivity and may be influenced by recall or interpretation bias, especially given the 24-month interval since surgery. Lastly, the absence of objective clinical assessments (e.g., uroflowmetry, pad tests, validated psychiatric diagnostics) limits the ability to correlate PROs with clinical endpoints. As a cross-sectional analysis at a single postoperative time point, causal inferences are not possible; a longitudinal study design would be necessary to better understand temporal dynamics and causality. Furthermore, due to the retrospective and cross-sectional design, causal relationships cannot be inferred. Moreover, potential changes in socioeconomic status, lifestyle habits, or psychological burden during the follow-up period were not captured, possibly influencing the reported outcomes.

Both open and robotic-assisted approaches were included in our cohort, reflecting the institutional transition in surgical technique during the study period, as well as routine clinical practice at a comprehensive cancer center. As data from a randomized phase 3 trial have shown no significant differences in long-term functional outcomes between the two techniques, and given that surgical approach was not the primary focus of this study, we did not perform a separate analysis comparing ORP and RARP (Yaxley et al. 2016). Nonetheless, the inclusion of both techniques may contribute to some heterogeneity in the functional outcome data.

Despite its limitations, our study provides relevant insights into the multidimensional determinants of patient-reported outcomes following radical prostatectomy. By examining socioeconomic, sociodemographic, and lifestyle factors alongside clinical variables, it highlights the importance of a holistic treatment approach that extends beyond purely medical considerations and acknowledges the individual circumstances of each patient. Our findings can support clinicians in identifying patients who may benefit from additional guidance or support—e.g. through psychosocial counseling, lifestyle interventions, or more personalized postoperative care planning. They also offer a realistic perspective on the information available to clinicians at the time of surgery and may inform the development of more tailored counseling strategies aimed at optimizing functional outcomes and long-term satisfaction. Ultimately, this study contributes to a growing understanding of how personalized, integrative care can enhance prostate cancer survivorship—moving beyond an organ-centered view toward a truly patient-centered approach.

## Conclusion

This study demonstrates that, beyond established pathological predictors, individual sociodemographic, socioeconomic, and lifestyle factors can influence long-term patient-reported outcomes following radical prostatectomy. While classical tumor characteristics were consistently associated with poorer functional and subjective results, psychosocial elements—such as perceived mental stress, partnership status, and physical activity—also emerged as relevant determinants. In contrast, socioeconomic status showed only selective associations, potentially moderated by the universal healthcare setting. These findings underline the value of a multidimensional, patient-centered approach in prostate cancer care. Incorporating psychosocial assessment and lifestyle counseling into perioperative management may improve recovery trajectories and help tailor follow-up strategies to individual needs.

## Data Availability

Data is provided within the manuscript. Additional data regarding multivariable analysis are available upon request.
